# The Effect of the Interactive Mobile Health and Rehabilitation System on Health and Psychosocial Outcomes in Spinal Cord Injury: Randomized Controlled Trial

**DOI:** 10.2196/14305

**Published:** 2019-08-28

**Authors:** Michael Alan Kryger, Theresa M Crytzer, Andrea Fairman, Eleanor J Quinby, Meredith Karavolis, Gede Pramana, I Made Agus Setiawan, Gina Pugliano McKernan, Bambang Parmanto, Brad E Dicianno

**Affiliations:** 1 Department of Physical Medicine and Rehabilitation University of Pittsburgh Pittsburgh, PA United States; 2 Department of Physical Medicine and Rehabilitation Penn State University Milton Hershey Medical Center Hershey, PA United States; 3 Human Engineering Research Laboratories Department of Veterans Affairs VA Pittsburgh Healthcare System Pittsburgh, PA United States; 4 Department of Occupational Therapy MGH Institute of Health Professions Boston, MA United States; 5 Department of Health Information Management School of Health and Rehabilitation University of Pittsburgh Pittsburgh, PA United States; 6 Department of Computer Science Udayana University Badung Indonesia

**Keywords:** cellular phone, emergency departments, hospitalization, mobile applications, pressure ulcer, rehabilitation, self-care, spinal cord injury, telemedicine, urinary tract infections

## Abstract

**Background:**

Individuals with spinal cord injury (SCI) are at risk for secondary medical complications, such as urinary tract infections (UTIs) and pressure injuries, that could potentially be mitigated through improved self-management techniques. The Interactive Mobile Health and Rehabilitation (iMHere) mobile health (mHealth) system was developed to support self-management for individuals with disabilities.

**Objective:**

The main objective of this study was to determine if the use of iMHere would be associated with improved health outcomes over a 9-month period. A secondary objective was to determine if the use of iMHere would be associated with improved psychosocial outcomes. Phone usage, app usage, and training time data were also collected to analyze trends in iMHere use.

**Methods:**

Overall, 38 participants with SCI were randomized into either the intervention group who used the iMHere system and received standard care or the control group who received standard care without any technology intervention. Health outcomes were recorded for the year before entry into the study and during the 9 months of the study. Participants completed surveys at baseline and every 3 months to measure psychosocial outcomes.

**Results:**

The intervention group had a statistically significant reduction in UTIs (0.47 events per person; *P*=.03; number needed to treat=2.11). Although no psychosocial outcomes changed significantly, there was a nonsignificant trend toward a reduction in mood symptoms in the intervention group compared with the control group meeting the threshold for clinical significance. Approximately 34 min per participant per month were needed on average to manage the system and provide technical support through this mHealth system.

**Conclusions:**

The use of the iMHere mHealth system may be a valuable tool in the prevention of UTIs or reductions in depressive symptoms. Given these findings, iMHere has potential scalability for larger populations.

**Trial Registration:**

ClinicalTrials.gov NCT02592291; https://clinicaltrials.gov/ct2/show/NCT02592291.

## Introduction

Spinal cord injury (SCI), an insult to the spinal cord that is most commonly traumatic, can be a life-changing diagnosis. In the United States, approximately 17,000 new injuries occur each year, resulting in a prevalence of 285,000 [1]. Beyond the acute injury, the impact to these individuals and the health care system is lifelong and costly. Incomplete tetraplegia, for example, accounts for 47.7% of SCI cases, entailing an average annual cost of care of US $1,102,403 for the first year and US $191,436 for subsequent years because of the multiple potential chronic complications of SCI [1]. While the primary characteristics of SCI include strength loss and sensory loss, these chronic complications can result in increased mortality, health care costs, treatments, and hospitalizations [1,2].

There are multiple complications that can occur after SCI. Two of the most common chronic complications are urinary tract infections (UTIs) and skin pressure injuries. UTIs occur because of neurogenic bladder [3] and bacteria entering the bladder during catheterization or catheterization not occurring on a consistent schedule, leading to retention of urine and growth of bacteria [4]. Diseases of the genitourinary system were the most common cause of death in SCI populations 40 years ago; however, the introduction of clean intermittent catheterization, treatment of bladder spasticity, and appropriate antibiotic treatment have resulted in a decrease in UTIs and related complications [2]. Skin pressure injury is a loss of oxygenation to the tissues caused by inadequate pressure relief that is triggered by poor sensation and impaired mobility [5]. Additional complications frequently associated with SCI include neurogenic bowel, pulmonary compromise, spasticity, and depression [2]. Depression is prevalent in the United States in 1 out of every 5 individuals with SCI as compared with 1 out of 20 people without disabilities [6].

Given the complex nature of SCI, people with SCIs and their families require extensive training and constant vigilance to prevent secondary complications [7]. Frequent communication is required between the patient and their medical team to prevent or treat complications [8]. As a result, the potential exists for using mobile health (mHealth) platforms to allow patients with SCI to proactively monitor their health and gain self-management skills to prevent complications.

Smartphones have become ubiquitous in American society, with over 77% of Americans owning a device in January 2018, compared with 55% in 2014 and 35% in 2011 [9]. Over 98.7% of individuals in developed countries and 70.4% in lesser developed nations have mobile broadband subscriptions [10]. However, the prevalence of mobile phone and mobile internet usage in the SCI population is not well studied. From 2010 to 2014, 46% of participants in the SCI Model Systems Centers reported using the internet on their phone [11]. It can be expected that smartphone usage among individuals with SCI will continue to increase in the future, as those who use smartphones preinjury will continue to use them post injury.

The use of mHealth platforms is likewise gaining popularity. New systems are being studied for many different types of patient populations and conditions, including older adults [12], chronic obstructive pulmonary disease [13], diabetes [14], and bipolar disorder [15]. The Apple App store and Google Play store each contain over 100,000 health and wellness smartphone apps, and developers are beginning to target chronically ill individuals, particularly those with diabetes, obesity, and hypertension [16]. Some apps used within rehabilitation populations have had positive impacts on mobility and self-management [17]. However, few randomized controlled trials using robust mHealth self-management interventions have been conducted.

**Figure 1:**
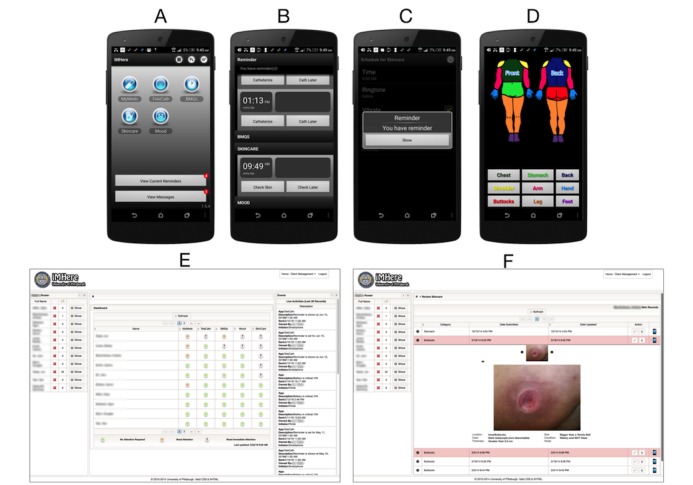
The iMHere interface. (a) Home screen with modules; (b) Screen for skin check reminder; (c) Reminder example; (d) Screen for charting a wound location; (e) Web-based portal used by coordinator to track iMHere users; (f) Example of a wound photo uploaded through the iMHere app. iMHere: Interactive Mobile Health and Rehabilitation.

The Interactive Mobile Health and Rehabilitation (iMHere) system (Figure 1) was developed to promote self-management for persons with disabilities and to facilitate communication between patients and their medical teams [18].

The first clinical trial of the iMHere system was conducted in the spina bifida (SB) population. It was found that higher usage of iMHere was associated with improved self-management skills, less caregiver assistance, and a decreasing trend in UTIs and emergency department (ED) visits [19]. Cost savings from the use of the system were estimated to be over US $15,000 per user per year. In a separate study, about 80% of individuals with SB and their caregivers felt the app would be easy to use and make a positive impact [19].

Many of the medical challenges in those with SB, including impaired mobility, neurogenic bowel, neurogenic bladder, insensate skin, polypharmacy, and depressed mood, are also present for those with SCI. Therefore, it was a natural extension to apply the iMHere system to the SCI population.

The goal of this study was to determine whether the use of the iMHere system would be associated with better health and psychosocial outcomes in patients with SCI. We hypothesized that the use of the app in addition to standard care would result in a larger magnitude of improvement in health outcomes (the primary outcome measures) and psychosocial outcomes (the secondary outcome measures) compared with a control group receiving standard care. Health outcomes were defined as the number of UTIs, number of pressure injuries, and number of ED visits and hospitalizations. Several specific survey outcomes were used as psychosocial metrics that described functional independence, quality of life, and mood.

An ancillary aim of the study was to determine whether any metrics related to phone use or app compliance impacted health and psychosocial outcomes. The time associated with managing the system and providing technical support was also evaluated.

## Methods

### Study Design

This study was a randomized control study. Baseline data were collected before randomization. Participants were randomized using a random number generator in Microsoft Excel (Microsoft). The control group received the standard of care in an outpatient physiatry SCI clinic and no technologic intervention. *Standard care* involves a patient being seen by an SCI-trained physician in an outpatient clinic on an intermittent basis, with follow-up as needed based on current health status, as determined by the physician. As part of standard care, the patient is able to call in to speak to a nurse, who can triage cases, offer recommendations, and pass concerns onto the physician. The physician can then decide whether any further evaluation is needed, which may include a clinic visit, recommendation to go to the ED, diagnostic testing, etc. The intervention group was given a Samsung Galaxy S5 smartphone with the iMHere app and received standard care in the same outpatient physiatry SCI clinic. Owing to the nature of the intervention, the study participants could not be blinded. However, the investigators who reviewed medical records and collected retrospective data and the individuals who conducted interviews were blinded to the participant group. It should be noted that the investigators who reviewed the electronic medical record were a physiatrist and physical therapist. Surveys were conducted by occupational therapy, medical, and nursing students. Consolidated Standards of Reporting Trials guidelines were used in the development of this study and in reporting the results. This study was registered in ClinicalTrials.gov, under registration number NCT02592291.

### Recruitment and Participants

This study was approved by the institutional review board of the University of Pittsburgh; all participants provided written informed consent. Participants were recruited from local physiatry-based SCI and assistive technology clinics. The inclusion criteria were (1) age 18 years and older; (2) diagnosis of SCI; (3) attends an outpatient physiatry clinic for SCI; and (4) lives in a community setting, rather than in a residential facility that provides care. The exclusion criteria were (1) diagnosis of severe intellectual disability or severe and persistent psychiatric illness and (2) actively participating in a concurrent wellness pilot program.

### Interactive Mobile Health and Rehabilitation System

The iMHere system (Figure 1) consists of an app used by the participant in the community and a Web-based portal for the clinician. The app includes several modules: (1) medication management, including medication administration reminders, the ability to upload photos of the medications, and customizable descriptions of the purpose for taking them; (2) urinary and bowel program reminders, with a system for reporting concerning symptoms; (3) skincare tracking with photo capabilities to monitor for pressure injuries and skin breakdown; (4) mood tracking with validated surveys; and (5) messaging, to communicate with a clinician [20]. The system has undergone multiple patient-centered design iterations to optimize the app for use by individuals with disabilities and their caregivers [19-25]. Intervention participants received 30 min of training to use the app. This involved app navigation, how to set up reminders, and how to record information in each module. The participants were told to use only the modules relevant to their recommended care regimen. After setting up the modules, the app would send them reminders in conjunction with their personal self-management routine. Participants were asked to respond to all reminders when they appeared on their device. If during their skin check reminder, they found a pressure injury, they were instructed to upload the location and a photo to the system. A physical therapist acted as the *wellness coordinator*, monitoring participant data using a Web portal and communicating with them electronically via their app.

### Health Outcomes

Health outcomes were collected by retrospective chart review for the 9 months before the study as well as for the 9 months during which each participant was enrolled in the study. Individual phone interviews with patients were used to verify or clarify information in the medical record. The number of UTIs and pressure injuries were both used because of the high incidence of such events in individuals with SCI [1]. Number of ED visits and hospitalizations were included because ED visits and hospitalizations both result in increased health care costs. The following health outcome measures were collected:

- Number of UTIs: Number of symptomatic UTIs with positive urine cultures that were subsequently treated with antibiotics.
- Number of pressure injuries: Number of unique episodes of skin breakdown, at least stage 2 or above, based on the National Pressure Ulcer Advisory Panel guidelines [26]. A unique pressure injury was defined either as a wound in a different area of the skin or in the same area with documented complete healing before reinjury.
- Number of ED visits: Number of encounters in the ED for any reason.
- Number of ED visits because of UTIs or pressure injury: Number of encounters in the ED specifically for UTI or pressure injury diagnosis, evaluation, or treatment.
- Number of hospitalizations: Number of admissions to the hospital for any reason.
- Number of hospitalizations because of UTIs or pressure injury: Number of admissions to the hospital specifically for UTI or pressure injury diagnosis, evaluation, or treatment.

### Psychosocial Outcomes

All participants were individually interviewed over the phone at baseline and every 3 months for 9 months, for a total of 4 interviews, using several psychosocial outcomes that are widely employed and validated to assess independence, mood, and quality of life in individuals with disabilities. The following questionnaires were used:

1. Canadian Occupational Performance Measure (COPM), which is a self-reported measure of self-care, productivity, and leisure [27].
2. Adolescent Self-Management and Independence Scale, which measures independence and self-management skills. The scale contains 10 items that measure independent living and 7 items that measure self-management skills and is valid for use in adults [28].
3. Beck Depression Inventory-II (BDI-II), which is a screening questionnaire that evaluates for symptoms of clinical depression, including guilt, self-blame, disappointment, satisfaction, and suicidal ideation [29].
4. Patient Assessment of Chronic Illness Care, which measures experience and satisfaction of chronic care [30].
5. World Health Organization Quality of Life Brief Instrument, which is a validated measure of perceived quality of life based on individual culture, values, and goals [31].
6. The physical independence domain of the Craig Handicap Assessment and Reporting Technique Short Form, which is a measure of perceived disability and independence [32]. This domain measures paid and unpaid caregiver hours on a 0 to 100 scale.

### Phone Usage, Interactive Mobile Health and Rehabilitation Usage, and Support Time

Usage statistics were recorded to gain a better understanding of how participants used their smartphone and the iMHere system to provide a potential explanation for differences in study results or rule out any potential confounding factors. Phone use habits were recorded using cellular phone bill data. The number of calls sent and received, text messages sent and received, and data used in megabytes were calculated for each participant. An iMHere compliance rate was also determined for each module and each participant by calculating the number of times the participant input data into each module, divided by the number of times the participant was prompted to input data into the module. If the participants input information more often than they were prompted, they were given a compliance rate of 1.

Toggle software (Tallinn) was used to record the amount of time that support was provided to individual participants. *Wellness Time* was defined as the time that the wellness coordinator spent triaging issues for participants or communicating with them about concerns. *Tech Support Time* was defined as the amount of time that each participant required for help with setting up the app, training, and any minor technical issues that arose during the study.

### Statistical Analysis

Sample size calculation was based on a previous study in which iMHere was used by participants with SB [25]. A moderate effect size of 0.30 was used and was based upon the primary outcome measures used in this study. A repeated-measures analysis of variance (ANOVA) yielded a sample size of 18 participants in each group for a power of 80%. Alpha values were set to .05 a priori*.*

The demographic information collected included gender, race, ethnicity, marital status, education, type of SCI, smoking status, assistance at home, and technology experience. The demographics of the intervention and control groups were compared to confirm that the randomization was effective using the Student *t* test, chi-square test, Fisher exact test, or Mann-Whitney test (Table 1). Baseline psychosocial outcomes were compared using the Mann-Whitney test.

**Table 1:** Participant demographics (N=19).

Demographic details		Intervention group	Control group
Age (years), mean (SD)		37.9 (13.4)	44.1 (15.3)
**Gender, n (%)**			
	Male	13 (68)	12 (63)
	Female	6 (32)	7 (37)
**Race, n (%)**			
	White	13 (68)	15 (79)
	Black	6 (32)	4 (21)
**Ethnicity, n (%)**			
	Hispanic	1 (5)	0 (0)
	Non-Hispanic	18 (95)	19 (100)
**Marital status, n (%)**			
	Single	12 (63)	11 (58)
	Not single	7 (37)	8 (42)
**Highest level of education, n (%)**			
	High school	11 (58)	10 (53)
	Higher education	6 (32)	8 (42)
**Completeness of injury, n (%)**			
	Complete	9 (47)	12 (63)
	Incomplete	10 (53)	7 (37)
**Functional status, n (%)**			
	Tetraplegia	8 (42)	9 (47)
	Paraplegia	11 (58)	10 (53)
Time since injury, mean (SD)		9.9 (8)	13.5 (11)
**Living status, n (%)**			
	Alone	2 (11)	2 (11)
	With others	17 (89)	17 (89)
**Student status, n (%)**			
	Student	3 (16)	1 (5)
	Not a student	16 (84)	18 (95)
**Smoking history, n (%)**			
	Smoker	8 (42)	6 (32)
	Nonsmoker	11 (58)	13 (68)
**Previous experience with smartphones, n (%)**			
	Yes	9 (47)	13 (68)
	No	10 (53)	6 (32)
**Previous experience with apps, n (%)**			
	Yes	9 (47)	11 (58)
	No	10 (53)	8 (42)

Primary health outcomes were tallied for the periods before study enrollment and during study enrollment and were compared pre- and postintervention using the Wilcoxon signed-rank test. A number needed to treat (NNT) analysis was performed for statistically significant and trending health outcomes. Generalized linear models with both fixed and random effects were used to evaluate changes in the secondary psychosocial outcomes over time. As this was an intention-to-treat analysis, participants with missing interview data were still included in the analysis.

Participants were split into high-usage phone users and low-usage phone users based on their average monthly general phone use habits. A high-usage phone user was defined as a participant who sent or received over 500 calls, sent or received over 1000 texts, or used over 3000 MB data using cellular connectivity. All other participants were classified as low-usage phone users.

The overall iMHere compliance rate for each participant was defined as the average compliance rate of all modules used by that participant. It should be noted that not all modules were used by all participants. A Student *t* test was performed to evaluate for an association between phone usage and overall compliance rate.

Intervention participants were divided into 2 groups: high overall compliance users (n=10) and low overall compliance users (n=9). A repeated-measures ANOVA was then used to evaluate whether there were any between-group differences in psychosocial measures with respect to overall compliance.

Statistical analyses of primary outcomes were performed using IBM SPSS Statistics for Windows (IBM Corp), and secondary outcomes analyses were performed using SAS version 9.4 (SAS Institute).

## Results

**Figure 2:**
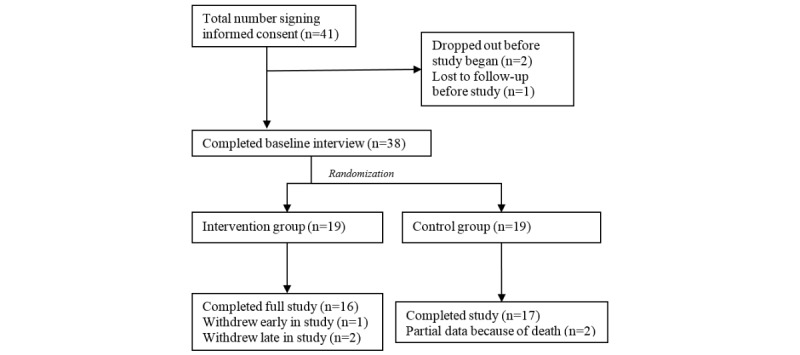
Flow diagram of patient enrollment and randomization.

A total of 41 participants were recruited to participate, and of those, 38 completed informed consent and baseline interviews. Figure 2 is a flow diagram demonstrating participant selection, randomization, and dropout. Tables 1 and 2 display participant demographics and baseline psychosocial outcome measures. No significant differences were detected at baseline between control and intervention groups.

**Figure 3:**
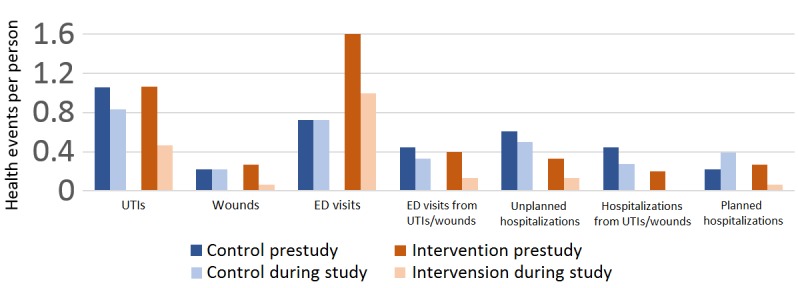
Health outcomes. ED: emergency department; UTI: urinary tract infection.

Figure 3 displays the incidence of health outcomes before and during the study period for the intervention and control groups. Participants in the intervention group experienced about half as many UTIs during the study period, when compared with the period before the intervention (*P*=.03). There was a reduction of 0.47 UTIs per person in the study group during the intervention compared with before the intervention. Such a reduction was not seen in the control group. No other primary outcome measures were found to change significantly in the intervention or control groups.

Table 3 presents the changes in psychosocial outcome measures in both groups during the study period. No statistically significant trends were seen between the intervention and control groups over time.

**Figure 4:**
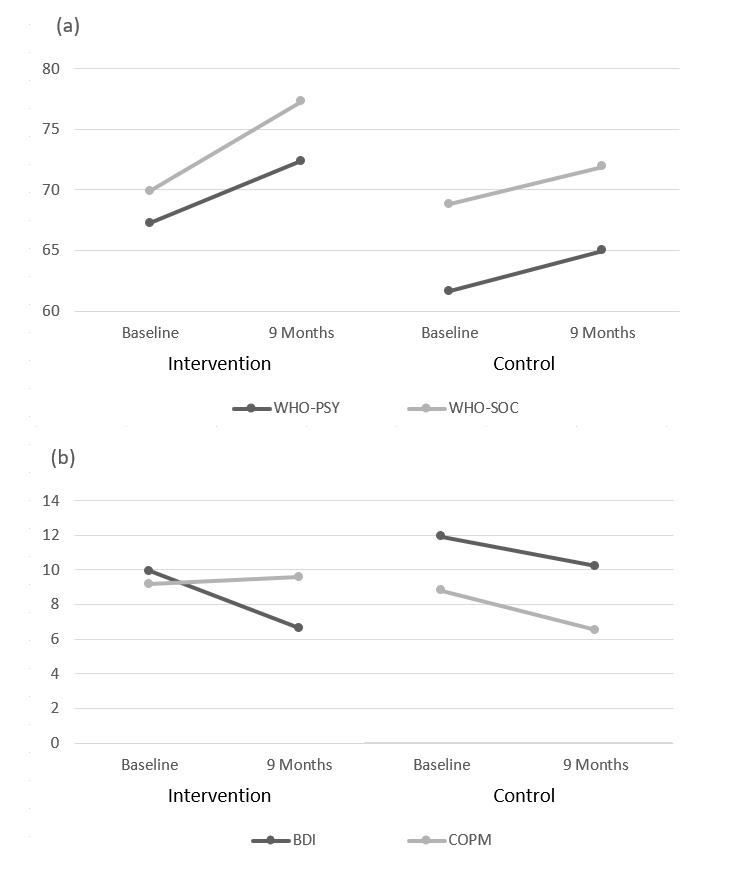
(a) Difference in World Health Organization Quality of Life subscores over time for intervention and control participants. (b) Difference in Beck Depression Inventory-II and Canadian Occupational Performance Measure scores over time for intervention and control participants. WHO-PSY: World Health Organization Psychiatric subscore; WHO-SOC: Social subscore; BDI: Beck Depression Inventory; COPM: Canadian Occupational Performance Measure.

Figure 4 demonstrates some of the general trends seen in Table 3 from baseline to 9 months for certain secondary outcomes.

Table 4 shows high-usage and low-usage phone users with corresponding overall iMHere compliance rates. There was no statistically significant difference in overall iMHere compliance rates between the high-usage and low-usage phone users (*P*=.41).

No statistically significant differences were seen in the 2 overall iMHere compliance rate groups with respect to changes in psychosocial outcomes (all *P* values were .45 or higher).

As shown in Table 5, approximately 34 min per month per participant was spent on providing wellness coordinator and technical support.

**Table 2:** Baseline comparison of psychosocial outcome measurements (N=19).

Outcome measure		Intervention, mean (SD)	Control, mean (SD)
Canadian occupational performance measure		8.59 (6.43)	8.58 (7.47)
**Adolescent Self-Management and Independence Scale-II**			
	Independence subscale	5.68 (1.08)	5.21 (0.98)
	Self-management subscale	5.86 (1.03)	5.70 (1.08)
	Total	91.06 (15.16)	86.21 (13.89)
Beck Depression Inventory-II		11.18 (8.65)	12.05 (10.79)
Patient Assessment of Chronic Illness Care		3.39 (0.83)	3.02 (0.54)
**World Health Organization Quality of Life**			
	Physical subscale	57.06 (12.21)	56.47 (12.01)
	Psychological subscale	64.06 (15.95)	62.05 (15.84)
	Social subscale	65.76 (24.96)	69.50 (24.27)
	Environment subscale	69.53 (18.71)	73.53 (13.44)
Craig Handicap Assessment and Reporting Technique Short Form		66.59 (37.36)	62.53 (29.00)

**Table 3:** Survey outcomes.

Outcome measure			Baseline, mean (SD)	3 months, mean (SD)	6 months, mean (SD)	9 months, mean (SD)
**Canadian Occupational Performance Measure**						
	Intervention		9.19 (7.91)	5.57 (9.10)	7.36 (12.12)	9.57 (14.07)
	Control		8.78 (7.64)	6.65 (5.48)	7.94 (8.03)	6.50 (6.45)
**Adolescent Self-Management and Independence Scale-II**						
	Intervention		90.56 (15.40)	91.29 (14.67)	89.29 (14.86)	90.79 (14.22)
	Control		87.39 (13.28)	87.18 (17.10)	89.31 (13.91)	90.06 (10.90)
**Adolescent Self-Management and Independence Scale-II**						
	**Independence subscale**					
		Intervention	5.62 (1.09)	5.62 (1.18)	5.50 (1.21)	5.54 (1.19)
		Control	5.33 (0.84)	5.37 (1.01)	5.46 (0.86)	5.43 (0.75)
	**Self-management subscale**					
		Intervention	5.88 (1.03)	6.06 (1.02)	5.54 (1.76)	6.62 (0.72)
		Control	5.69 (1.11)	5.62 (1.40)	5.82 (1.02)	5.99 (0.92)
**Beck Depression Inventory** **-II**						
	Intervention		9.94 (6.74)	8.07 (5.65)	4.86 (5.87)	6.64 (4.53)
	Control		11.89 (11.08)	12.35 (13.30)	11.38 (9.92)	10.19 (9.61)
**Patient Assessment of Chronic Illness Care**						
	Intervention		3.54 (0.68)	3.34 (0.85)	3.24 (0.97)	3.44 (0.78)
	Control		3.04 (0.56)	3.31 (0.70)	3.09 (0.68)	3.09 (0.74)
**World Health Organization Quality of Life**						
	**Physical subscale**					
		Intervention	59.06 (12.84)	63.07 (13.12)	61.29 (11.42)	60.43 (12.33)
		Control	56.11 (12.26)	54.94 (13.36)	53.31 (14.20)	59.56 (7.99)
	**Psychological subscale**					
		Intervention	67.25 (14.38)	70.71 (10.30)	72.79 (13.13)	72.36 (9.21)
		Control	61.67 (16.21)	60.47 (16.42)	60.75 (15.07)	65.00 (13.66)
	**Social subscale**					
		Intervention	69.88 (25.37)	77.64 (12.97)	78.14 (13.69)	77.29 (16.82)
		Control	68.82 (24.84)	68.00 (22.27)	71.81 (22.32)	71.94 (23.22)
	**Environment subscale**					
		Intervention	72.69 (19.98)	75.14 (15.60)	73.21 (17.24)	77.43 (15.36)
		Control	73.44 (13.83)	75.47 (13.70)	77.44 (14.61)	81.81 (12.16)
**Craig Handicap Assessment and Reporting Technique Short Form**						
	Intervention		74.00 (34.78)	75.86 (34.95)	72.57 (32.83)	74.86 (27.60)
	Control		62.22 (29.81)	66.59 (34.32)	70.00 (28.13)	66.00 (25.17)

**Table 4:** Phone and Interactive Mobile Health and Rehabilitation (iMHere) app usage (N=19).

ID	Phone usage group	Calls sent and received, n	Text messages sent and received, n	Data used (MB)	Overall iMHere app compliance (%)
1	High	992	1955	3022	98
3	Low	1	3	2817	18
7	High	0	11	3234	46
8	High	34	2963	1565	45
9	High	4105	47,689	11,853	39
11	Low	14	58	1676	57
12	High	234	2445	25,563	42
14	Low	15	9	1079	30
16	Low	5	34	1097	98
18	Low	83	20	1097	51
19	Low	57	38	902	23
20	High	4463	3429	63,074	49
25	High	2120	8173	99,191	88
30	Low	81	62	317	18
31	Low	313	68	1464	74
32	Low	179	161	2641	42
40	High	3495	1589	21,268	85
47	High	2663	18,596	21,364	47
49	High	1362	8640	28,556	20

**Table 5:** Support time (N=19).

Contact time descriptions	Wellness time	Tech support time	Total time
Total contact time for 19 participants in 9 months, hours:minutes	49:00	56:23	105:23
Average contact time per participant in 9 months, hours:minutes (SD)	2:08 (2:08)	2:27 (2:44)	4:35 (3:35)
Average contact time per participant per month, hours:minutes (SD)	0:14 (0:14)	0:16 (0:18)	0:34 (0:24)

## Discussion

### Principal Findings

This study contributes to the literature by demonstrating successful use of an mHealth platform in individuals with SCI. This study demonstrated a significant reduction in UTIs (one of the primary outcomes) in those who used iMHere over time when compared with the control group. Given the reduction in UTIs during the intervention, 0.47 fewer UTIs per person, the NNT to prevent 1 UTI is 2.11. As not all intervention participants used the catheterization module, the study was not powered to determine whether the use of this specific module was associated with the reduction in UTIs. One explanation for reduction in UTIs aside from the use of the catheterization module is increased general health awareness and improved self-management that resulted from using the app in general. Unfortunately, it was challenging to determine the financial implications of these findings as there is a lack of literature examining health care costs associated with the treatment of UTIs in an outpatient setting in individuals with chronic SCI. However, in similarly aged adults with SB, the cost to treat a UTI was found to be approximately US $511 per event [33]. Although no other significant changes were seen in health outcomes over the study period, the other 6 primary outcome measures also decreased in the intervention group, which was a trend also observed in a similar study in the SB population [25]. More studies are warranted to determine whether larger patient cohorts might result in significant changes in these variables.

Of the secondary outcomes, the reduction in depressive symptoms based on BDI-II was the closest to approach significance between groups. The decrease over time in BDI-II in the intervention group, an average of 3.3 points or 33% (3.3/9.94), was twice that of the control group. Previous research has suggested that a decrease in BDI-II score of 17.5% may be clinically significant because it correlates with an individual *feeling better*. Therefore, this change could be considered clinically significant. It should be noted that the clinical significance of the magnitude of change depends upon an individual’s initial score [34]. Lower initial scores, meaning a person has fewer depressive symptoms, may require smaller changes in BDI-II score for an individual to *feel better*. This further supports the suggestion of clinical significance in the intervention group as the BDI-II was lower at baseline. As not all intervention participants used the mood module, the study was not powered to determine whether the use of this specific module was associated with the reduction in BDI-II score. Many of the control group participants used smartphones in everyday life, and those in the intervention group who used their phones more did not have better mood outcomes than those who used their phones less. These 2 findings suggest that the communication afforded by the phone itself was not solely responsible for this change. It is possible that iMHere’s ability to facilitate communication with the health care team, participants’ increased awareness of their own mood symptoms, or other improvements in health may have impacted mood positively. Notably, a review by Thota et al confirmed that collaborative care models that use case managers to connect patients, primary care physicians, and mental health professionals provide a supportive care network that empowers people with depression to take a self-management role in their own care [35].

As our study was underpowered for the secondary outcomes, it is not unexpected that the other psychosocial outcomes were not statistically significant. A descriptive analysis reveals that there are some trends (as shown in Figure 4). There is a larger general improvement in World Health Organization Quality of Life psychological subscore at 9 months, which makes sense in the context of the improvement in BDI-II. There is also a markedly higher social subscore. It should be noted that Figure 4 also shows that COPM improved slightly in the intervention group and decreased substantially in the control group, suggesting overall that participants in the intervention group perceived their self-care, productivity, and leisure to be maintained after 9 months, whereas those in the control group had declined in this perception. We postulate that a larger study cohort may have allowed us to detect significant changes.

Overall, iMHere compliance rates were not related to psychosocial outcomes or the amount of phone usage. This contrasts with findings from a previous study in SB in which more frequent users of iMHere had positive changes in self-management skill and amount of caregiver assistance needed [25]. One possible explanation for the contrasting findings between studies is that those with tetraplegia in this study may not have been able to reduce the need for hands-on care even if they did gain small improvements in knowledge about self-management. Although significant accessibility features have been implemented for individuals with tetraplegia and other impairments [22-24], it is also possible that users with paraplegia were able to use the system more proficiently. In addition, because we used billing data to calculate usage data, we may have underestimated the usage of individuals who primarily used Wi-Fi.

The integration of mHealth support into outpatient care depends in part on the requirements for staff effort [23]. This study demonstrated that wellness and technical support requires on average approximately 34 min per user per month. This information may be useful when scaling mHealth interventions to larger populations.

### Study Limitations

Some limitations of this study warrant discussion. First, this study was powered to detect statistical differences in the primary outcomes related to health and not the secondary psychosocial outcomes. As a result, even though there was a trend toward improved psychosocial outcomes in the intervention group, the study was unable to find statistical significance in these trends. Future studies with larger sample sizes are planned to help with this issue. One potential confounder was that additional contact with study staff for wellness coordination or technical support may have had an impact on outcomes in the intervention group. Although a small amount of support was provided in person or via phone, the majority of contact was virtual, through the mHealth system. To evaluate whether such contact may have offset other types of contact, we conducted a post hoc analysis. However, no statistically significant changes were seen within or between groups with respect to the number of outpatient visits, phone calls to the clinic, and hospital health portal messages. This was likely because the number of instances of these occurrences was low on average. It is also possible that the use of iMHere shifted use for some nonurgent issues from the ED to the outpatient setting. It is important to note that the control group may also have had more contact with clinicians through ED visits and hospitalizations. More work will be needed to understand which aspects of an mHealth delivery system are most beneficial to outcomes and to provide more insight into how a self-management app might affect health care utilization and service delivery. A second limitation is the small sample size, which may have reduced our ability to detect changes in outcome measures with lower effect sizes. A larger population with greater usage levels of individual modules may have enabled us to do a subgroup analysis to determine if there was a correlation between individual module usage and health outcomes. Third, the inclusion of individuals with tetraplegia may have resulted in a ceiling effect of how much improvement can occur in some outcomes such as self-management given that they will still likely rely on caregiver assistance. Future studies will be aimed at evaluating outcomes using mHealth support for caregivers [8,36]. We are also investigating the addition of more accessibility features to support users with tetraplegia such as voice control. Fourth, the iMHere system has multiple features, but not all features were relevant to all users. Larger studies will be needed to evaluate the individual effects of different aspects of the system. Finally, fully functional smartphones were provided for study purposes, but some individuals also used their personal phone, which may have reduced the usage of study phones. The new version of iMHere (2.0) operates cross-platform and can now be used on personal devices.

### Future Directions

Concurrent work on iMHere has produced a subsequent version (iMHere 2.0) with additional features. A new smartphone app will support family or formal caregivers and interface with the client app. A personal health record and additional modules have also been built to support community integration, physical activity, nutrition, goal setting, and education [21,37]. Future work will be conducted to evaluate the implementation of these features into clinical workflows, translation to larger and different disability populations and clinical settings, and interfacing with other electronic health systems.

### Conclusions

Overall, the use of the iMHere mHealth platform resulted in a statistically significant reduction in UTIs over time compared with the control group. On the basis of an NNT analysis, 2.11 users were needed to prevent 1 UTI. There was also a decrease in several other outcome measures (eg, symptoms of depression), which trended toward, but did not reach, statistical significance. Approximately 34 min per participant per month is needed to provide education, care coordination, and technical support through this mHealth system, thus suggesting scalability.

## Abbreviations

ACL: Administration for Community Living
ANOVA: analysis of variance
BDI-II: Beck Depression Inventory-II
COPM: Canadian Occupational Performance Measure
ED: emergency department
HHS: Health and Human Services
iMHere: Interactive Mobile Health and Rehabilitation
mHealth: mobile health
NIDILRR: National Institute on Disability, Independent Living, and Rehabilitation Research
NNT: number needed to treat
SB: spina bifida
SCI: spinal cord injury
UTI: urinary tract infection

## Appendix

Multimedia Appendix 1 CONSORT-EHEALTH Checklist (V 1.6.1).
